# Chiroptical Switches: Applications in Sensing and Catalysis

**DOI:** 10.3390/molecules17021247

**Published:** 2012-01-31

**Authors:** Zhaohua Dai, Jennifer Lee, Wenyao Zhang

**Affiliations:** 1 Department of Chemistry and Physical Sciences, Pace University, 1 Pace Plaza, New York, NY 10038, USA; 2 Department of Chemistry, New York University, 100 Washington Square East, New York, NY 10003, USA

**Keywords:** chiroptical switches, molecular recognition, sensor, chiral analyte, achiral analyte, asymmetric catalysis

## Abstract

Chiroptical switches have found application in the detection of a multitude of different analytes with a high level of sensitivity and in asymmetric catalysis to offer switchable stereoselectivity. A wide range of scaffolds have been employed that respond to metals, small molecules, anions and other analytes. Not only have chiroptical systems been used to detect the presence of analytes, but also other properties such as oxidation state and other physical phenomena that influence helicity and conformation of molecules and materials. Moreover, the tunable responses of many such chiroptical switches enable them to be used in the controlled production of either enantiomer or diastereomer at will in many important organic reactions from a single chiral catalyst through selective use of a low-cost inducer: Co-catalysts (guests), metal ions, counter ions or anions, redox agents or electrochemical potential, solvents, mechanical forces, temperature or electromagnetic radiation.

## 1. Introduction

The study of molecular recognition, especially chiral recognition, is of considerable significance. A characteristic of many enzyme systems is their ability to distinguish between enantiomers in catalytic reactions. Chemosensors based on molecular recognition are of great importance because of their potential for monitoring intracellular components, pollutants, chemical and biological warfare agents, and other analytes. Detection of non-racemic molecules in extraterrestrial objects and determination of their handednesswould suggest that complexity on the scale required for life is present outside of our planet and contribute to the investigation of origin of life. The US Food and Drug Administration and similar agencies worldwide have been favoring the marketing of enantiomeric forms of drugs, whose syntheses generally require enantioselective chiral catalysts. Equally important is the development of quality control protocols for the determination of absolute configuration and enantiomeric purity. Chiroptical sensors with the necessary sensitivity are needed to discriminate small enantiomeric excesses. The prospect of advancing chemical sensor technology, modeling electron-transfer processes in biological systems, and producing new catalysts has led to considerable interest in the design and synthesis of receptor molecules that contain chiral centers. A better understanding of the interactions operating in chiral recognition is helpful in such development. One recent interest in molecular recognition is switchable materials with responsive chemical, electrical, optical, and other properties. Among these, chiroptical switches could play a key role in the development of next generation sensors and catalysts.

One important challenge for the design of efficient sensors is to engineer sensors capable of selectively detecting multiple analytes, with each analyte prompting different signals. Sensors based on chiroptical switches could prove to be able to tackle such a challenge. For such a chiroptical sensor, each different analyte would induce a different chiral supramolecular assembly, producing different chiroptical responses. Chiroptical sensors, when the sensing-material is chiral, can be employed to analyze achiral species, too. One advantage of using chiral sensors is that they form complexes with achiral species with defined configuration and chirality can be utilized to control the stereochemistry of the resultant complexes, thus modulating binding selectivity [1,2]. In addition, they can yield chiroptical, anisotropic information on top of isotropic information so that the ligand can not only signal the presence of an achiral species, but evaluation of both properties can allow identification and quantification of the species.

One highly sought-after goal for the design of efficient selective catalysts for asymmetric catalysis is to control the chirality of the catalytic ligand itself using supramolecular chemistry, which can be achieved by dynamically inducing a stereogenic element through a readily available low-cost inducer, chiral or achiral [3]. Achieving switchable control over catalysts and sensors will further our understanding of such systems and empower us to exploit their full potential. Triggers or handles of control include chemical inputs, solvents, electrochemical means, temperature, and irradiation (polarized or not). For such triggers to induce different catalytic outcome, they should be “recognized” or “sensed” by the main ligand. In this sense, the sensing and catalytic behaviors of chiroptical switches are closely related. Switchable catalytic behavior is then expected based on their switchable recognition of different stimuli. Unexpected inversions in asymmetric catalysis, which are reviewed in a recent article that provide important information related to this review [4], are generally out of the scope of this review. This review will mainly cover chiroptical switches that are used as senors and/or catalysts and will be organized by stimuli input mechanism. More extensive coverage on chiroptical switches can be found in other review articles [5,6,7,8,9,10,11,12,13,14,15].

Chiroptical spectroscopic measurements are powerful tools for the characterization of materials and outcomes in these fields, and may include any form of polarized light measurement [16]. A recent book chapter [13] on metal-base dynamic stereochemical systems is organized by their chiroptical responses: Optical rotation [17], ORD [18], ECD, VCD [19], FDCD/CPE and CPL [20]. We provide brief explanations of the chiroptical spectroscopic terms used in this paper in the assessment of chiroptical switches as listed in Table 1.

**Table 1:** Terms and definitions in chiroptical spectroscopy.

**Type**	**Acronym**	**Definition**
Optical rotation	α	The rotation of linearly polarized light as it travels through certain non-racemic materials.
Optical rotatory dispersion	ORD	The variation in the specific rotation of a substance with a change in the wavelength of light. May be used to determine the absolute configuration of metal complexes, for example.
Circular dichroism	CD	A form of spectroscopy based on the differential absorption (Δε) of left- and right-handed circularly polarized light (LCP and RCP) in non-racemic molecules.
Exction-coupled circular dichroism(Exciton Chirality)	ECCD	Circular dichroism coming from an exciton couplet: Two chromophores presenting in a molecule in close proximity to one another, who interact and give rise to differentiation of the energies of the transitions.
Fluorescence-detected CD	FDCD	Circular dichroism derived from the differential emission of light from a sample excited with LCP and RCP radiation when the analyte is chiral, fluorescent, and the quantum yield is the same for both circularly polarized components.
Diffrentialcircularly polarized fluorescence excitation	CPE(ΔF)	The differential emission of light from a sample excited with LCP and RCP radiation. The quantum yield does not have to be the same for both circularly polarized components
Circularly polarized luminescence	CPL	The anisotropic emission of circularly polarized light originated from non-polarized excitation.

## 2. Chemically Triggered Chiroptical Switches

### 2.1. Chiroptical Switches Triggered by Chiral Guests

**Figure 1:**
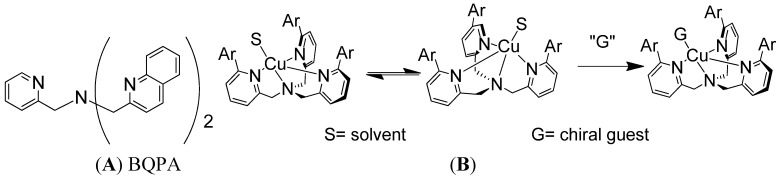
(**A**) Structure of BQPA. (**B**) Racemate Cu(II) complex of BQPA analogues reacts with chiral amine, resulting in a helix twist in one direction, which produces an ECCD spectrum.

Supramolecular conformational bias based on host-guest complexes includes nonempirical CD approaches for the determination of the absolute configuration of primary amines [21]. One of the much sought-after goals has been to develop a system that could report absolute configuration or enantiomeric excess without derivatization or separation. The reagents in non-derivatization methods can potentially be recycled and used multiple times, which makes it greener and more cost effective. A non-derivatization method for the determination of absolute configuration of various chiral amines has been developed in the Canary group using the conformational racemate complex **1**, [Cu(BQPA)](ClO_4_)_2_ (BQPA = *N,N*-bis[2-quinolyl]methyl-*N*-[2-pyridyl]methylamine) (Figure 1A) [22]. The analyte guest chiral amine would associate with the Cu(II) complex to form a ternary complex. The chirality of the guest molecule would induce the helical twist of the three aryl arms to be predominately in one direction (Figure 1B), and such a directional twist from chromogenic groups would elicit a characteristic ECCD response upon absorbing polarized light. Left-handed guests induce left-handed propeller twist, yielding a positive ECCD couplet.

**Figure 2:**
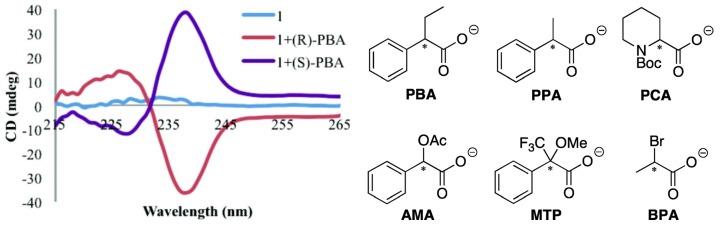
**Left**: CD spectra of Cu(II) complex of BQPA (complex **1**, 0.5 mM) by itself and with each enantiomer of PBA (1.0 mM) in 75% MeCN/H_2_O with 20 mM HEPES buffer at pH 7.4 [23]. **Right**: Structures of PBA and other analytes. Reproduced by permission of the American Chemical Society.

Anslyn and Canary *et al.* recently pushed this method further forward. Enantiomeric complexes were created upon binding of the enantiomers of the carboxylate guests to this achiral host Cu(BQPA)](ClO_4_)_2_, and the sign of the resultant CD signal allowed for determination of the configuration of the studied guest (Figure 2) [23]. The difference in magnitudes and shapes of the CD signals, in conjunction with linear discriminant analysis (LDA), allowed for the successful identification of the guest. In a related study, Anslyn *et al.* developed a rapid assay of enantiomeric excess of chiral diamines using racemic copper(I) or Pd(II) complexes of some achiral ligands using an instrument interfaced to a robotic 96-well plate to rapidly and conveniently measure the CD spectra of a compound library [24,25].

**Figure 3:**
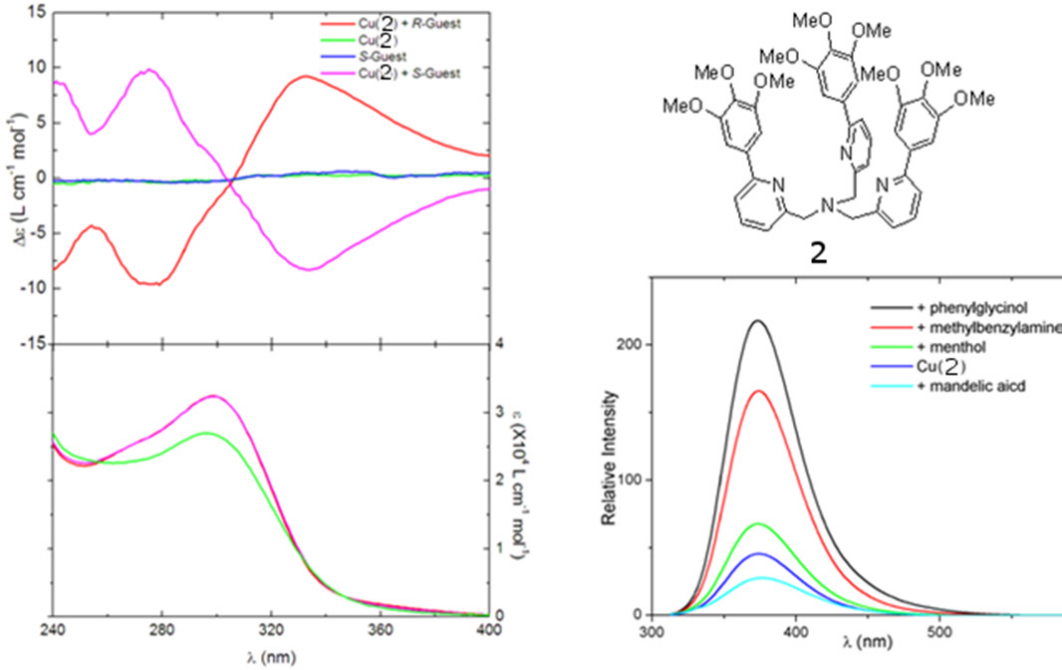
(**A**) Circular dichroism (top) and UV (bottom) spectra of [Cu(**2**)]^2+^ complexed with chiral guests 2-phenylglycinol. (**B**) Fluorescence modulation of [Cu(**2**)]^2+^ by selected small molecule guests [26]. Reproduced by permission from the author of reference [26].

However, the relatively short absorption and emission wavelength of the quinoline chromophores presents several limits. First, light sources of short wavelength endanger the stability of organic materials. The interaction between the short-wavelength chromophore and the short-wavelength aromatic rings in many chiral amines can lead to spurious results. The ECCD signal with short-wavelength chromophores tends to be small in the non-derivatization methods. The absorption maxima of short-wavelength chromophore are below the cut-off of may solvents, limiting their application. Such chromophores are often undesirable fluorophores with short emission wavelength and low quantum yield so that they cannot be used in FDCD or CPE, which will be discussed later. Long-wavelength chromophores with high extinction co-efficients and high quantum yields might be very promising in addressing these problems. Recently, compound **2** (Figure 3), a derivative of tris(pyridylmethyl)amine (TPA), was synthesized [26]. It is found that chiral amine, such as (*S*)-2-phenylglycinol or (*R*)-2-phenylglycinol can form stable ternary complexes with the Cu(II) complex of ligand **2**. The *R* configuration guest also induces an exciton-coupled CD spectrum as an exact mirror image to that of the *S* configuration guest. In this compound, the three aromatic arms are identical (Figure 3A). Its Cu(II) complexes can potentially produce three ECCD couplets in a chiral environment, according to the additive rule of ECCD. Therefore, the overall amplitude of the ECCD spectra shown in Figure 3A is bigger than when [Cu(BQPA)](ClO_4_)_2_, which can produce only one ECCD couplet, is used. More interestingly,although Cu(II) quenches the fluoresence of the free ligand **2**, addition of guest molecules containing amino group, such as phenylglycinol and methylbenzylamine, can recover the fluorescence of the free ligand (Figure 3B).

Chiral rigidified piperidine and quinuclidine analogs of BQPA were examined for asymmetric recognition of amino compounds by cyclic voltammetry and fluorescence. A Cu(II) complex of a piperidine analog discriminated the enantiomers of some chiral amines and amino-alcohols, giving differences in electrochemical potential for diastereomeric complexes [27]. Protonated piperidine and quinuclidine analogs were able to differentiate the two enantiomers of certain amino-alcohols by fluorescence spectroscopy.

Alkane-bridged or pentanediol-linked bis(zinc porphyrin) hosts, referred to as a “zinc porphyrin tweezer” [28], undergo syn-to-anti conformational switching upon complexation with external ligands and unidirectional screw formation based on the stereochemistry of the chiral guests. A variety of substrates including aromatic amines, cyclic and acyclic amines, amino esters, amides, and cyclic amino alcohols have been examined by this and similar approaches and the method has been generalized to substrates with various potential points of ligation, including monodentate compounds [29,30,31] Holmes *et al.* used such a tweezer for the determination of absolute configuration and enantiopurity of amphetamines and methamphetamines, expanding their application to the field of forensic science [32]. Other bridged bis(zinc porphyrin) systems [33,34,35,36] have also been reported as chiroptical sensors and have been recently reviewed [14]. Holmes and Balaz at al recently used unbridged achiral anionic nickel(II) meso-tetra(4-sulfonatophenyl) porphyrin for the highly sensitive and specific chiroptical detection of condensed left-handed Z-DNA in the presence of canonical right-handed B-DNA [37]. Other chiroptical sensors [20,38,39,40] for chiral guests have also been recently reviewed [13,14,41].

**Figure 4:**
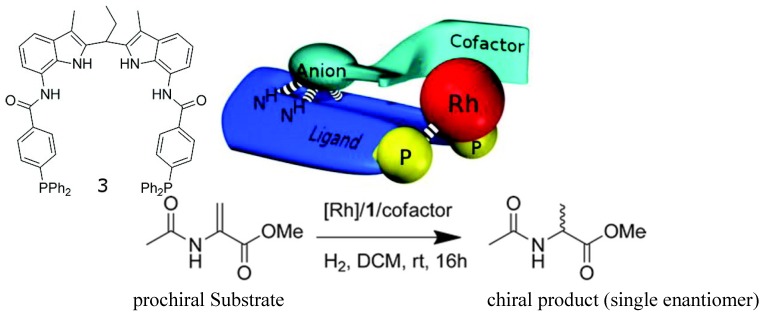
Structure of ligand **3** (top left) and the general concept of cofactor controlled enantioselective catalysis (top middle), the best cofactor (top right), and the catalyzed hydrogenantion reaction (bottom) [42]. Reproduced by permission of the American Chemical Society.

Such guest-induced chiral switches can find application in asymmetric catalyst. An achiral rhodium complex of an achiral bisphosphine ligand (Figure 4) equipped with a binding site for the recognition of chiral anion guests has been reported [42]. Upon binding small chiral guests-cofactors (chiral organic acids or amino acids)-the rhodium complex becomes chiral and can be used for asymmetric catalysis of the hydrogenation of prochiral substrates. The best cofactors among a library lead to hydrogenation catalysts that form the products with high enantioselectivity (*ee*’*s* close to 99% when the co-factor is the one shown in Figure 4, top right). Interestingly, high *ee* was obtained when a mixture of 12 cofactors were used, indicating that the complex based on the best cofactor dominates the catalysis.

### 2.2. Chiroptical Switches Triggered by Achiral Guests

#### 2.2.1. Chiroptical Switches Triggered by Achiral Organic Compounds

**Figure 5:**
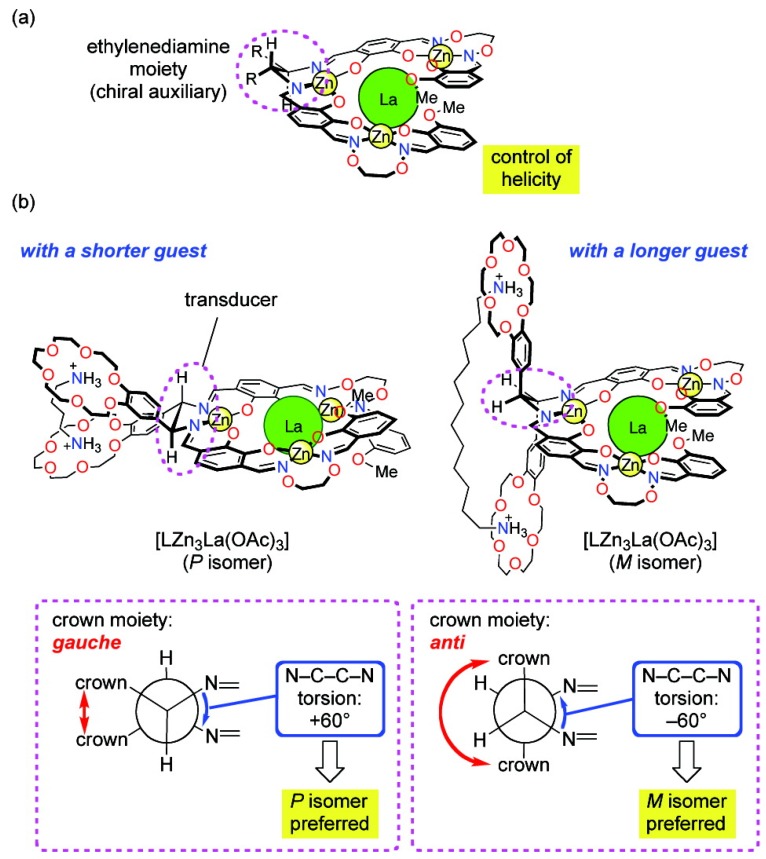
A helical tetranuclear complex bearing two benzocrown moieties and a small chiral auxiliary (an ethylenediamine moiety) can change helicity depending on the size of an achiral guest [43]. Reproduced by permission of the American Chemical Society.

A helical tetranuclear complex bearing two benzocrown moieties and a small chiral auxiliary (an ethylenediamine moiety) was prepared as a novel molecular leverage for helicity control (Figure 5). It is found that if the N-C-C-N torsion angle of the ethylenediamine moiety is changed from +60^○^ to −60^○^, the handedness of the helical scaffold of the tetranuclear complex moiety can be inverted. Short alkanediammonium guests H_3_N^+^(CH_2_)*_n_*NH_3_^+^ (*n* = 4, 6, 8) preferentially stabilized the *P*-helical isomer of the complex, while the longer guest H_3_N^+^(CH_2_)_12_NH_3_^+^ caused a helix inversion to give the *M*-helical isomer [43].

#### 2.2.2. Chiroptical Switches Triggered by Metal Cations

**Figure 6:**
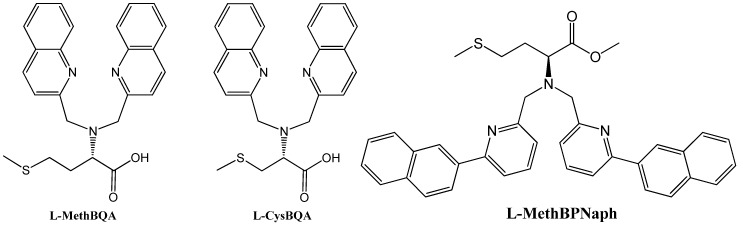
Structures of L-MethBQA, L-CysBQA and a recently synthesized analog L-MethBPNaph.

**Figure 7:**
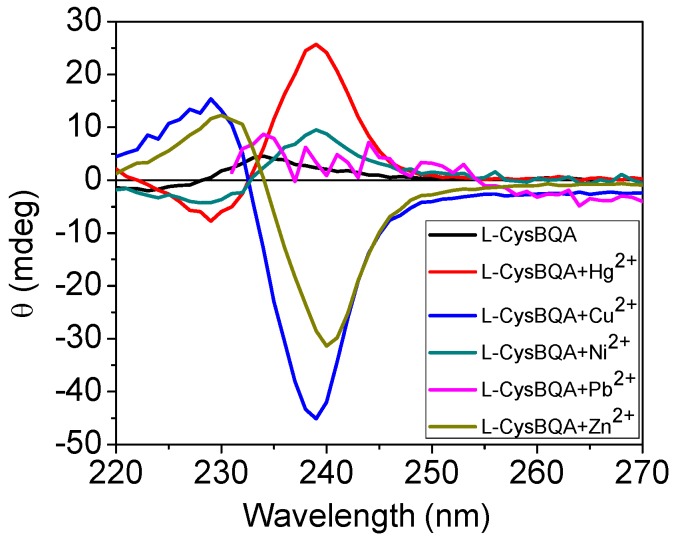
Circular dichroism response of 10 μM L-CysBQA to 50 μM HgCl_2_, Cu(ClO_4_)_2_ and Zn(ClO_4_)_2_, Pb(ClO_4_)_2_ and Ni(ClO_4_)_2_ respectively, in 30:70 acetonitrile/water [44]. Reproduced by permission of Wiley.

Many metal ion-induced switches have been reported [45,46,47,48,49] and reviewed [11,13,14]. Recently, two multi-mode Hg(II) sensors, L-MethBQA and L-CysBQA (Figure 6) [44], were obtained by fusing methionine or *S*-methyl cysteine, into a bis-quinolyl amine based chiral podand scaffold. Quinolyl groups serve as the fluorophore and possess nitrogen lone pairs capable of chelating metal ions. Upon exposure to Hg^2+^ or Zn^2+^ these sensors show signal enhancement in fluorescence. However, Cu^2+^ quenches their fluorescence in 30:70 acetonitrile/water. L-CysBQA complexes with Hg^2+^, producing an exciton-coupled circular dichroism spectrum with the opposite sign to the one that is produced by Cu^2+^ or Zn^2+^ complexation (Figure 7).

**Figure 8:**
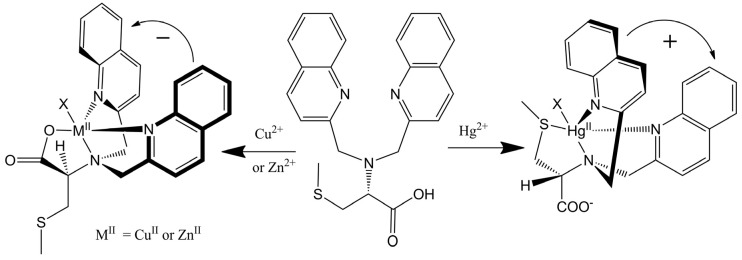
(*R*)**-***N*,*N*-Bis(2-quinolylmethyl)-*S*-methyl cystein (L-CysBQA) complexes with Cu^2+^/Zn^2+^ and Hg^2+^. The chiral center of the amino acid dictates the orientation of the quinoline chromophores via a gearing mechanism as illustrated. The transition dipoles in the quinolines in the two complexes invert in the sense of absolute orientation and therefore give opposite ECCD spectra [44]. Reproduced by permission of Wiley.

This remarkable differentiation of Hg^2+^ from Cu^2+^ and Zn^2+^ can be explained by the structures of the CD active products, which are illustrated in Figure 8. Tetradentate metallochelates form involving the Cu^2+^ ion, the tertiary amine, the two quinolines, and the carboxylate. The stereocenter of the *S*-methyl cysteine arm dictates the orientation of the quinoline groups by a “gear” with the methylenes of the achiral arms such that a propeller forms from the planar carboxylate and quinoline groups. The Hg^2+^ complex gives a propeller complex with the opposite configuration due to the preference of Hg^2+^ for coordination by the sulfur atom. As shown in Figure 8, for the sulfide to come proximal to the metal center, the amino acid arm must pivot about the C-N bond. This inverts the gearing, and therefore the orientation, of the quinoline moieties, leading to an exciton coupled CD with the opposite sign. Although crystals of L-CysBQA complexes with these metal ions have yet to be obtained, crystal structures of Zn^2+^ and Cu^2+^ complexes of L-MethBQA and other similar compounds, which show strong exciton couple CD in organic solvents, showed that these metal ions coordinate with the carboxylate instead of the sulfide [50,51,52]. NMR spectra of the Cu^+^ complexes and other evidence demonstrated Cu^+^-S coordination [50,53], which indicates that Hg^2+^ should coordinate the sulfur atom since like the soft Cu^+^, the soft- Hg^2+^ should prefer the soft sulfur.

**Figure 9:**
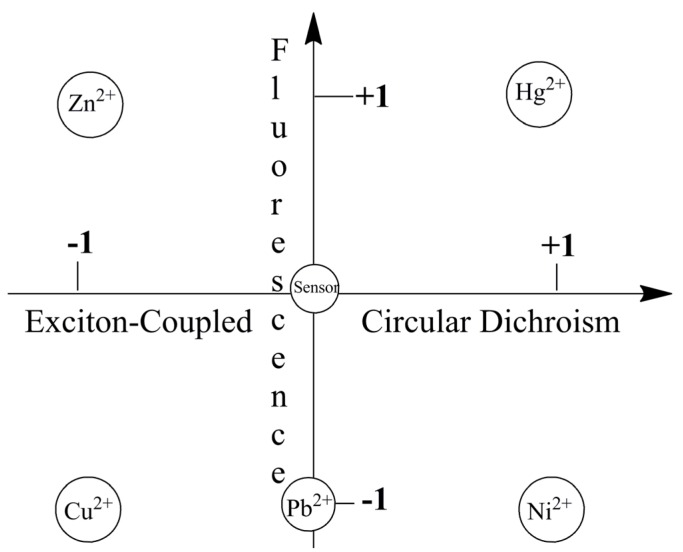
Chiroptically enhanced fluorescence detection and differentiation of different metal ions by L-CysBQA through pattern recognition [44]. Reproduced by permission of Wiley.

Figure 9 visually highlights metal ion sensing by the chiroptical fluorescent sensor L-CysBQA through both fluorescence enhancement and anisotropic absorption distinguishing, for example, Hg^2+^ (enhanced fluorescence with strong positive exciton coupled CD), Zn^2+^ (enhanced fluorescence and strong negative exciton coupled CD), Cu^2+^ (strong negative exciton coupled CD but quenched fluorescence), Ni^2+^ (strong positive exciton coupled CD but quenched fluorescence) and Pb^2+^ (quenched fluorescence but no exciton coupled CD). L-MethBQA offers similar advantages. These results further illustrate that recognition involving both isotropic and anisotropic detection tools may be utilized to maximize the information transmitted by a single sensor molecule [54].

Interestingly, a pyridyl analog L-BPNaph (Figure 6, right) shows fluorescence enhancement as well as significant spectral red-shift in emission upon exposure to HgCl_2_ [55]. However, its CD spectrum does not change upon addition of HgCl_2_, ZnCl_2_, CuCl_2_, Zn(ClO_4_)_2_, Cu(ClO_4_)_2_, Ni(ClO_4_)_2_ or Pb(ClO_4_)_2_. Further investigation is in progress in our laboratory.

Consideration of potential chiroptical properties of such chiral fluorescent tripodal ligands opens possibilities to develop new analytical methods to improve sensitivity or contrast. For example, when the ligands or their complexes are fluorescent, sensing events can be monitored by fluorescence-detected circular dichroism (FDCD). FDCD measures the difference in fluorescence intensity upon excitation by left and right-circularly polarized light [56]. Typically, the two channels of raw FDCD data, which correspond to the difference in emission (F_L_ − F_R_, F_L_, F_R_ = fluorescence with left and right circularly polarized excitation, respectively) and the total emission (F_L_ + F_R_) resulting from differential absorption of left- and right-circularly polarized light, are converted to CD spectrum by established methods. An adaptation of the FDCD technique can provide a unique and powerful new strategy for sensor applications by using the ΔF (ΔF = F_L_ − F_R_) [56], component of FDCD data directly. This method was named differential circularly polarized fluorescence excitation (CPE) to distinguish this new approach from traditional FDCD [57]. This is different from circularly polarized luminescence (CPL) because CPL is the differential spontaneous emission of left and right circularly polarized light from non polarized light excitation and reflects the structural properties of the excited state, while CPE is mainly due to the differential absorption of left- and right-circularly polarized light. CPE is still an indirect reflection of the structural properties of the ground electronic state when fluorescence polarization is negligible. The theoretical basis of this approach can be derived from established Equation (1):

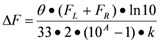
(1)

Fluorescence intensity is proportional to the numbers of photos absorbed as is expressed in Equation 2, where Φ_F_ is fluorescence quantum yield:

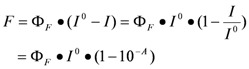
(2)

If ΔA = A_L_ − A_R_ ≤ 0.1 and ΔA/A ≤ 0.1, total emission (F_L_ + F_R_) should be proportional to fluorescence induced by non-polarized light, *i.e.*:
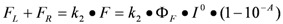

where k_2_ is another instrumental constant. Therefore Equation (2) can be simplified as shown in Equation (3), where K is a constant, which incorporates all other constants and Φ_F_ is the fluorescence quantum yield:

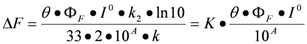
(3)

Materials with higher ellipticity θ and higher fluorescence quantum yield Φ_F_ will lead to an even larger ΔF. Substances lacking either fluorescence or CD properties will not be observed. The contrast in ΔF signal between a sample with both a large quantum yield and a large CD and a sample with both a small quantum yield and a small CD will be much larger than the contrast in either fluorescence or CD signals.

**Figure 10:**
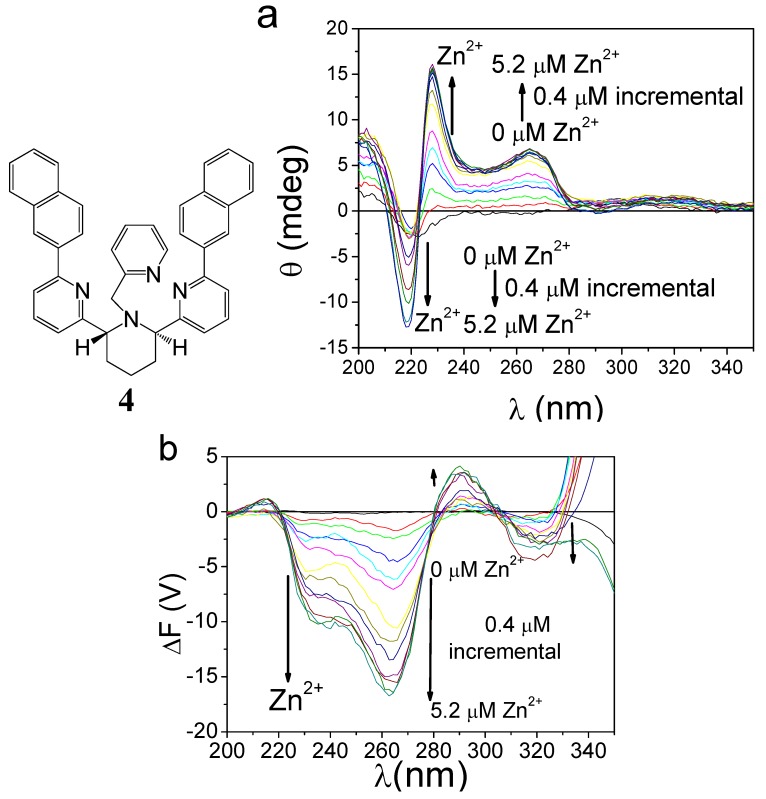
Spectral responses (a, CD; b, CPE) of 4.8 μM *(R,R)-***4** to 0-5.2 μM Zn(II) in acetonitrile [57]. Reproduced by permission of the American Chemical Society.

In conventional FDCD measurements where the purpose is structure determination, the voltage applied to the detector varies in each experiment, ensuring a maximal total fluorescence output signal voltage from the detector of 1.0 V to get the best sensitivity. However, in studies that aim to follow the relationship between the dynamic stereochemistry in metal complex and metal concentration by CPE (ΔF), appropriate sample concentration and electrical voltage are applied so that fluorescence signals were suitable for FDCD measurements (highest total fluorescence diode of the FDCD gave a maximum around 1.0 V for the saturated zinc complexes), and the voltage, together with the rest of the conditions, will be kept constant during each titration experiment. If we follow the conventional method in which the voltage is adjusted each time metal ions are added or removed, then the ΔF signal will no longer reflect the concentration of zinc. Therefore, to measure a sample’s response to a foreign species, all experimental conditions must be kept unchanged. Shown in Figure 10 are the CD and CPE (ΔF) responses of a chiral piperidine compound (**4**) to Zn^2+^ [57]. Apparently, FDCD and CPE may be used to monitor the chirality switching when a chiroptical sensor is exposed to different metal ions.

When fluorescence emission is polarized as in CPL, CPE would reflect the difference in both absorption and emission of left- and right- circularly polarized light, thus becoming potentially more sensitive to external stimuli than both FDCD and CPL. CPE’s great potential has been exemplified by recent development employing polarized light excitation to probe the chiroptical responses from a single bridged triarylamine helicene molecule embedded in polymer films [58]. The distributions of dissymmetry (g) parameters by analysis of signals from pure *M*- and *P*-type diastereomers are almost perfect mirror images of one another, each spanning a range of both positive and negative values. In addition, a well-defined structure in the histogram of dissymmetry parameters suggestive of specific molecular orientations at the polymer interface was observed. These single molecule results highlight strong intrinsic circular dichroism responses that can be obscured by cancellation effects in ensemble measurements of a randomly oriented bulk sample. Therefore, CPE might be useful in the single molecule detection of mercury in a complicated biological matrix such as tissues and organs.

#### 2.2.3. Chiroptical Switches Triggered by Achiral Anions

There are several interesting examples of chiroptical switches triggered by interaction with anions [13,14,59,60,61] Interest in such systems is heightened because supramolecular recognition of anions developed later than that of cations, and the abundance of potential anionic analytes in biology and other areas.

**Figure 11:**
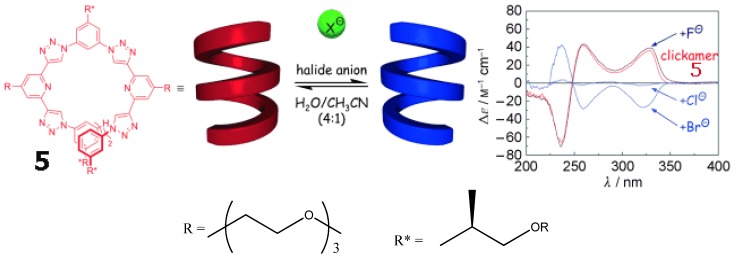
Optical response of clickamer **5** (8 × 10^−6^ M, 75 vol% water in acetonitrile, 25 °C) to various halide ions: KF, KCl, and KBr (all at neutral pH) as well as HCl [62]. Reproduced by permission of Wiley.

A “clickamer” **5** (Figure 11, left) was prepared trough click chemistry [62]. Its backbone consists of embedded helicogenic motifs based on alternating triazole and pyridine moieties. It displays two complete turns and hence a significant number of stabilizing π-π stacking contacts. At higher water content, the more intense short-wavelength absorption maximum of the clickamer backbone is associated with an intense bisignate signal exhibiting positive exciton chirality with a null at 246 nm as well as another bathochromically shifted, positive signal at 260 nm (Figure 11, right). The addition of HCl resulted in an exact inversion of the CD signal. The effect of various halide anions on the CD spectra was investigated at neutral pH. Whereas addition of fluoride ions led to a slight increase in CD intensity relative to the neat oligomer, chloride ions caused an inverted CD of smaller intensity. Bromide ions induced an inverted CD spectrum of higher intensity. All CD spectra display an isodichroic point at 248 nm and that the positions and relative intensities remain constant, reflecting an inversion of the same helical conformation.

**Scheme 1:**
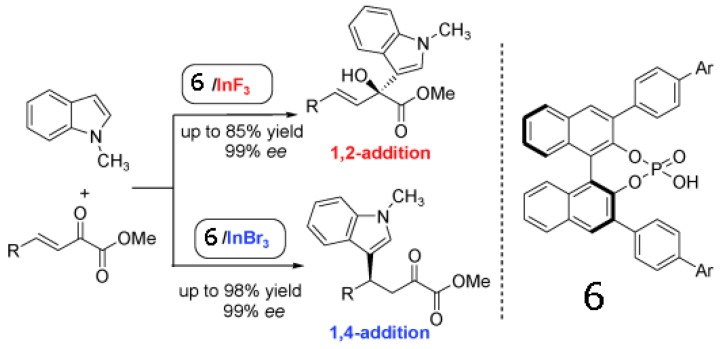
Counter-anion switches regioslectivity in enantioselctive Friedel-Crafts alkylation of indoles [63]. Reproduced by permission of Wiley.

Luo group present an asymmetric binary acid catalyst that synergistically combines a chiral phosphoric acid (compound **6**) and an indium halide salt to achieve regioselective Friedel-Crafts alkylation of indoles with β,γ-unsaturated α-keto esters (Scheme 1) [63]. A simple swap of counteranions of In(III) from fluoride to bromide or chloride switches the regioselectivity from 1,2- to 1,4-addition. Regardless of the regioselectivity, excellent reactivity and enantioselectivity in both cases were obtained. These results underline the potential importance of counteranions in tuning stereoselectivity.

### 2.3. Solvent-Triggered Chiroptical Switches

**Figure 12:**
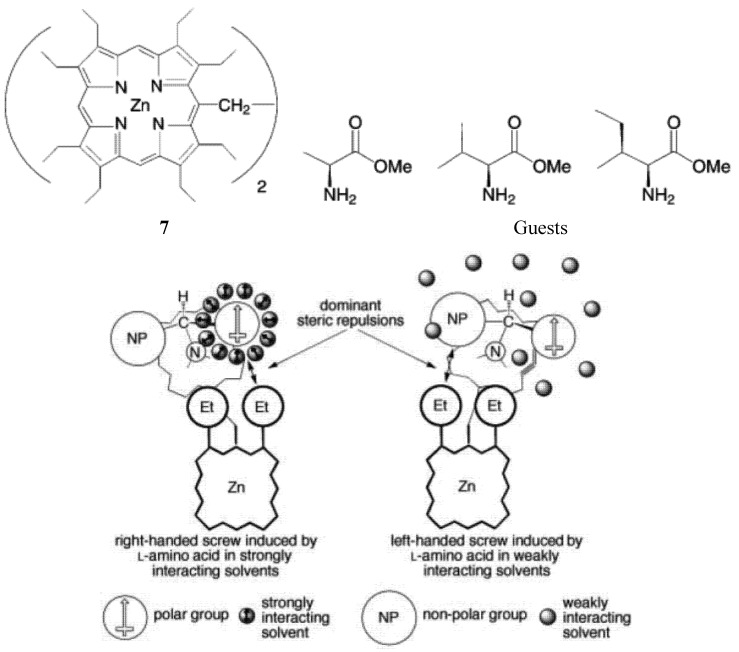
Solvent effect on the mechanism of supramolecular chirality induction in complexes between a zinc porphyrin scissor (**7**, top left) and amino acid derivatives (top right), in strongly and weakly interacting solvents (the ligand coordinated to the upper porphyrin has been omitted for clarity) [64]. Reproduced by permission of Wiley.

As mentioned earlier, “zinc porphyrin tweezers” have been used to probe the stereochemistry of the chiral guests. Interestingly, Borovkov, and Yoshihisa Inoue found that achiral solvents affects the supramolecular chirality of the supramolecular complex between an ethane-bridged bis(zinc porphyrin) host **7** and chiral guests (methyl esters of some amino acids) (Figure 12) [64]. It was found that in polar media, the solvent molecules are able to interact with the ester group of the amino acid derivatives (the guests) through strong dipole-dipole forces. Consequently, a structurally organized solvent shell surrounding the polar substituent is formed. Nonpolar alkyl substituents are unable to interact with polar solvents in such a manner. As a result, the relative “effective sizes” of the competing ligand substituents can be changed and even inverted. When the size of a nonpolar side-chain substituent is equal to, or slightly larger than, that of the polar C-terminus ester group, an increase in the “effective size” of the polar group in polar solvents, such as CHCl_3_, ethyl acetate, CH_2_Cl_2_ and 1,2-dichloroethane, can tip equilibrium towards the formation of the right-handed screw in the guest-host complex (when guest has *S* configuration) (Figure 12). This helical orientation corresponds to positive-induced chirality, and is in accordance with the exciton chirality method. In contrast, in nonpolar solvents such as *n*-hexane, cyclohexane, benzene and CCl_4_, because of the negligible solvent-solute interactions, the dominant steric repulsion takes place between the larger side chain and ethyl group of the neighboring porphyrin ring. This interaction induces a corresponding equilibrium shift towards the formation of the left-handed screw, which corresponds to negative exciton chirality.

A solvent-triggered molecular switch reported recently by Nitschke [65] was reasoned to be dominated by hydrogen bonding effects. A weakly polar solvent such as dichloromethane only weakly interacts with the hydroxyl groups of the ligand, allowing for intramolecular hydrogen bonding. Such hydrogen bonding rigidifies the structure and locks the complex into the *P* conformation. Polar solvents such as DMSO interact strongly with the ligand hydroxyl groups to push them apart, leading to a preference for the *M* conformation. There are many solvent triggered chiroptical switches involving lanthanide complexes of chiral ligands [66,67], which are reviewed recently [13].

Increasing attention has focused on polymer-based chiroptical sensors and chiral catalysts [8]. They typically feature easy separation from the sensing/reaction mixture and the possibility of re-use of the sensor/catalyst. The Yashima group synthesized and studied many chiral polymers, which contributes significantly to this field. This group recently extensively reviewed their work and other groups’ work on helical polymers [9]. In some molecular designs for polymer-based chiral catalysts, chiral induction relies solely on the chiral main chain structure of the polymers, which is quite attractive in that fine-tuning or even a dramatic change of the chiral reaction environment would be possible because of the large conformational change of the polymer secondary structures. One of the most attractive triggers for such switches is solvent. The helicity of poly(propiolic esters) can be switched by CHCl_3_ and hexane [68].

**Figure 13:**
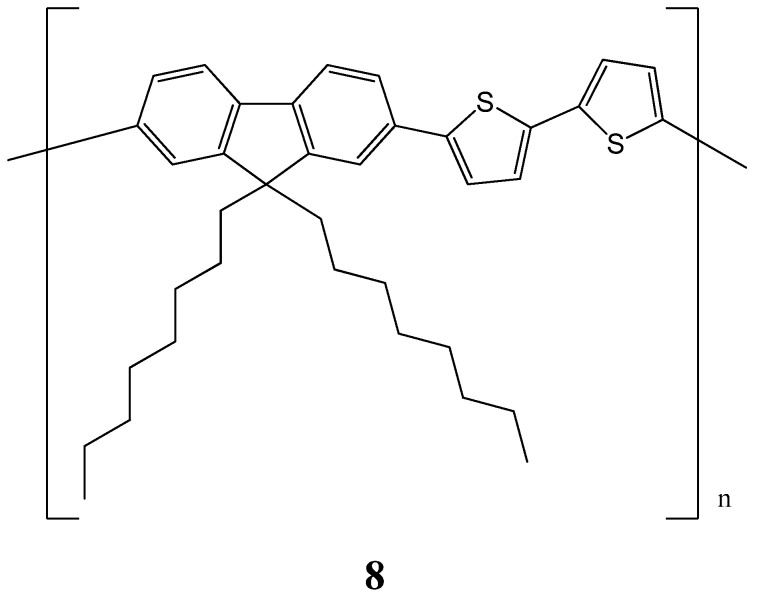
Structure of polymer **8** [69].

Solvent chirality transfer using (*S*)- and (*R*)-limonenes allowed for the successful production of optically active poly[(9,9-di-*n*-octylfluorenyl-2,7-diyl)-alt-bithiophene] (**8**) (Figure 13) particles with circular dichroism (CD) and circularly polarized luminescence (CPL) properties [69]. The most intense CD and CPL spectra were produced when the CD-silent **8** was dissolved in (*S* or *R-*limonene)-chloroform-methanol mixture [2.0:0.3:0.7 (v/v/v)]. A bisignate CD band around 510 and 390 nm clearly emerged in the π-π* transitions of **8**. The sign of an almost mirror-image CD band profile was determined by the limonene chirality. When methanol was replaced by ethanol, the CD amplitude was greatly diminished by a factor of 50. More strikingly the sign of the CD was reversed, suggesting an inversion of the twisting direction in the π-π∗ stacks. The changes in CPL were similar.

**Figure 14:**
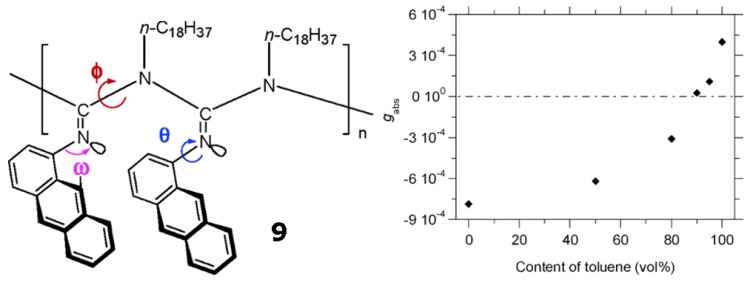
Kuhn’s dissymmetry ratio, *g*_abs_ (=Δε/ε), at 380 nm of **9 **in toluene/THF at 25 °Cclearly demonstrates the chiroptical inversion driven by the change in solvent composition between toluene and THF. The chiroptical inversion occurs around 90% content of toluene [70]. Reproduced by permission of the American Chemical Society.

Novak *et al*. synthesized a stereoregular poly[*N*-(1-anthryl)-*N′*-octadecylguanidine] (**9** in Figure 14) with a relatively narrow polymer dispersity index [70]. At room temperature, polymer **9** shows a positively signed Cotton effect in toluene, but negative ones in THF and chloroform, respectively. The chiroptical switching takes place around the toluene content of 90% (vol) in the mixed toluene/THF solvents (Figure 14). This is the first example of chiroptical switching phenomenon occurring in a helical polymer possessing no chiral moieties in the polymer chains. This reversible chiroptical switching phenomenon occurs by reorientation of anthracene rings relative to the chain director. Possible molecular motions leading to such reorientation include the N-C bond rotations (*φ*) in the backbone, *N*-C_Ar_ bond rotations (*θ*) in the side chains, and the imine configuration interconversions (*ω*).

**Figure 15:**
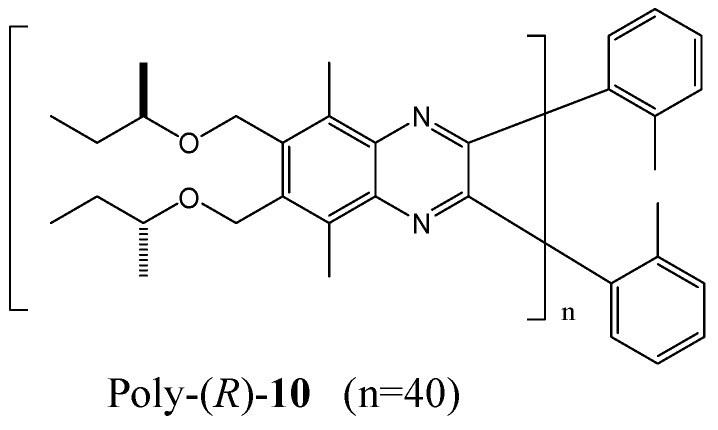
Structure of poly-(*R*)-**10** in [71].

The Suginome group has established that poly(quinoxaline-2,3-diyl)s bearing chiral (*R*)-2-butoxy side chains (for example, poly-(*R*)-**10** in Figure 15) adopt pure right- and left-handed helical senses in CHCl_3_ and 1,1,2-trichloroethane (1,1,2-TCE) respectively [71]. It is assumed that the helix switch may depend on solvent induced conformational changes of the chiral side chains. Although each chiral group may gain a very small energy for one helix, an accumulation of 40 monomer units was sufficient to form an almost perfect *M*-helix. CD measurements for poly-(*R*)-**10** (40 mer) in various solvents revealed that the *P*-helical conformation was strongly preferred in CHCl_3_, CH_2_Cl_2_, THF, and 1-butanol, while moderate *P*-helix induction was observed in 1,1,1-thichloroethane, 1-chlorobutane, and 1-bromobutane. In valeronitrile, 1,2-dichloroethane, and 1,3-dichloropropane, moderate M-helix induction was observed.

**Figure 16:**
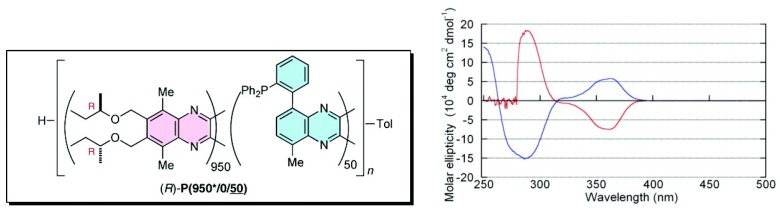
CD spectra of (*R*)-P(950*/0/50) in chloroform (blue line) and in a 1,1,2-trichloroethane/toluene (2/1) mixture (red line) [72]. Reproduced by permission of the American Chemical Society.

**Scheme 2:**
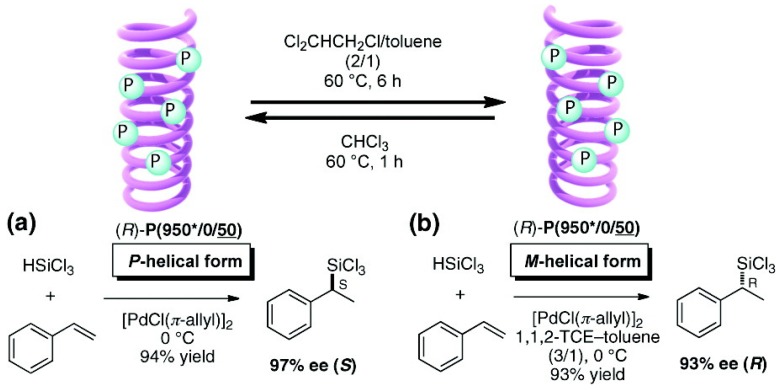
Asymmetric hydrosilylation using chirality-switchable (*R*)-P(950*/0/50) adopting (**a**) *P*-helical form or (**b**) *M*-helical form [72]. Reproduced by permission of the American Chemical Society.

Suginome *et al*. reported that high-molecular-weight (ca. 1,000 mer) variants of PQXphos bearing chiral side chains exhibited not only much higher enantioselectivity in asymmetric hydrosilylation of styrenes but also a solvent-dependent switch of its own helicity (Figure 16) and the chirality of the product (Scheme 2) [72]. CD spectrum showed that in chloroform, copolymer (*R*)-P(950*/0/50) adopted an almost pure *P*-helical structure and showed excellent enantioinduction (97% *ee*) for the (*S*)- product in asymmetric hydrosilylation of styrene (Scheme 2). When the same polymer was dissolved in a mixture of 1,1,2-trichloroethane/toluene (2/1), gradual inversion of the helical sense was observed by CD measurement(Figure 16). After 6 h at 60 ^○^C, it turned almost entirely to a *M*-helix, which afforded 93% *ee* for the (*R*)- product in the catalytic hydrosilylation of styrene in 1,1,2-trichloroethane/toluene (Scheme 2). In this way, the controlled production of either enantiomer from a single chiral catalyst is achieved through selective use of solvents.

**Scheme 3:**
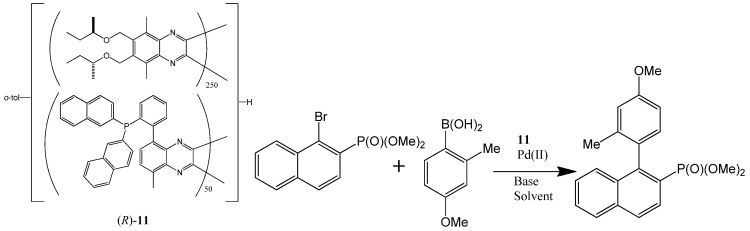
The enantioselectivity of a Suzuki coupling catalyzed by the Pd(II) complex of a chiral **polymer** (*R*)-**11** (left) can be switched solvents [73]. Reproduced by permission of Wiley.

Suginome *et al*. also showed that helically chiral PQXphos serves as a highly enantioselective ligand in the Suzuki-Miyaura coupling reaction to form axially chiral biarylphosphinic esters (Scheme 3) [73]. The high molecular weight 1,000 mer-based ligand (*R*)-**11** bearing bulky P(2-nap)_2_ (R = 2-naphthyl) groups was obtained from its toluene solution by precipitation with MeOH. This ligand possessed pure right-handed (*P*) helical structures, which catalyzed one Suzuki-Miyaura coupling reaction (shown in Scheme 3) in THF/H_2_O to afford an axially chiral biarylphosphinic ester of (−)-(*S*) configuration with 98% *ee*. The helical sense of this ligand was found to be switchable by solvents. The pure left-handed helical polymer ligand (*M*)-(*R*)-**11** can be obtained by heating a 1,1,2-trichloroethane/THF solution of (*P*)-(*R*)-**11** for 24 h. The resultant left-handed helical polymer (*M*)-(*R*)-**11** was used as a ligand in the same reaction with 1,1,2-trichloroethane added in the solvent, generating the (+)-(*R*) product with 90% *ee*. Although other similar ligands’ helicity could be switched by solvents too, the catalytic outcomes of such switches were not mentioned.

**Scheme 4:**
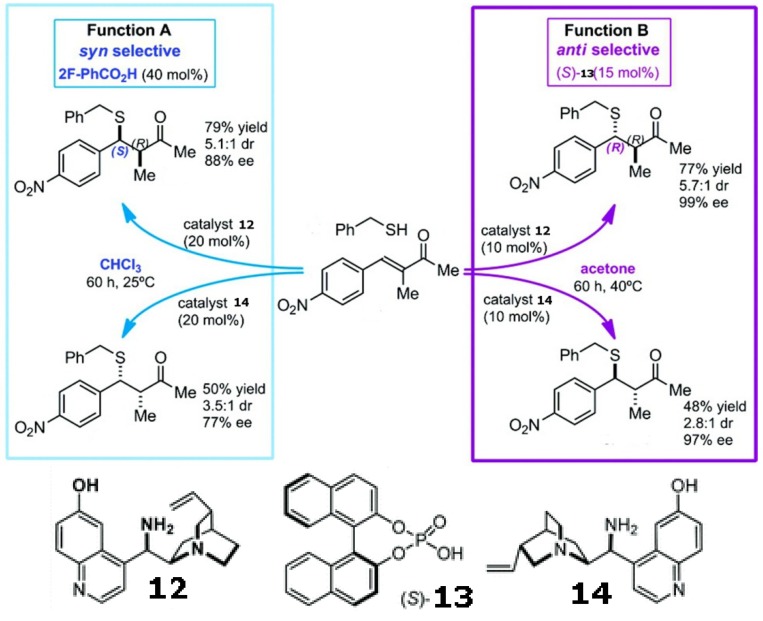
Diastereoselectivity can be switched by solvents and additives using a single catalyst in the asymmetric conjugate addition of alkyl thiols to α-substituted α,β-unsaturated ketones [74]. Reproduced by permission of the American Chemical Society.

Mirror image products can be provided by the enantiomeric pair of a chiral catalyst. However, modulating the enforced sense of diastereoselectivity using a single catalyst was only recently demonstrated in the asymmetric conjugate addition of alkyl thiols to α-substituted α,β-unsaturated ketones using a quinuclidine derivative with a pendant primary amine (shown in Scheme 4). Solvents, together with acidic additives, switch the sense of the catalyst’s diastereoselection, thereby affording either the syn or anti product with high *ee* and *de * [74].

### 2.4. Redox-Triggered Switches

Changes in CD spectra of redox-sensitive material are also very useful. Redox based chiroptical switches, including iron [75], copper [76], ruthenium [77] and platinum [78] complexes of a host of chiral ligands, have been recently reviewed [10,12,13,14] Tripodal ligand metal complexes offer particularly rich stereodynamic behavior, especially when coupled with exciton chirality analysis.

**Figure 17:**
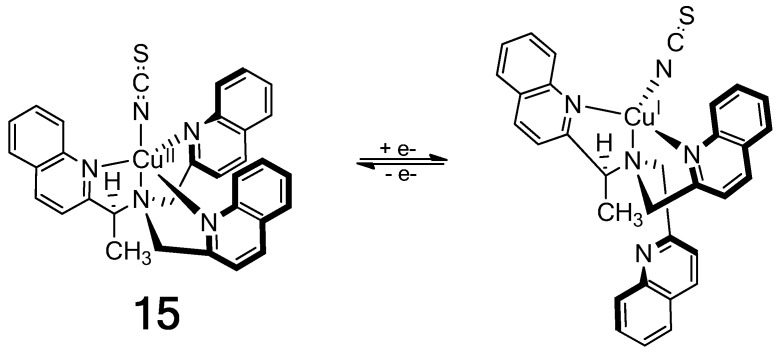
On/off chiroptical molecular switch: One-electron reduction of a Cu^2+^ complex results in dissociation of an arm of the chiral tripodal ligand [79]. Reproduced by permission of Wiley-VCH.

The Cu(II) complex of a chiral tris(quinoline) ligand (**15**) in Figure 17 gives an exceptionally intense ECCD spectrum that results from the additive effect of three ECCD couplets in one molecule [79]. Reduction by ascorbate to the Cu(I) complex in the presence of strongly coordinating thiocyanate ion prompts the dissociation of one achiral quinoline arm, which results in a much weaker (1/3 of the Cu(II) complex) ECCD spectrum originated from only one ECCD couplet. Oxidation of the Cu(I) complex with persulfate restores the characteristically intense ECCD spectrum of the Cu(II)-complex.

One related copper complex contains two chiral carbon centers within a piperidine ring [80]. In the Cu(I) oxidation state, the ligand adopts a relatively stable cyclohexane chair conformation, with two equatorial and one axial substituent. One pyridine moiety is placed remotely from the metal ion, which is accommodated by the lower coordination number of the Cu(I) ion. The Cu(I) complex did not give an exciton chirality spectrum. In the Cu(II) state, strong binding to the higher-coordination number ion brings all three pyridines into association, which forces the piperidine to adopt a higher energy chair with two axial and one equatorial substituents, showing the largest ECCD amplitude of any complex in this series.

Redox changes (Cu(I)/Cu(II)) also gave large changes in twisting power of these complexes to torque a nematic into a cholesteric liquid crystalline phase [81]. The handedness of the induced cholesteric phase was related to the stereochemistry of the complex.

**Figure 18:**
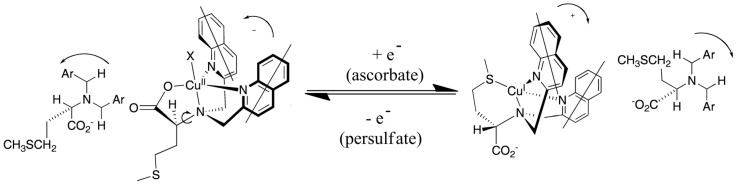
Redox-induced inversion of helicity as a result of the presence of gearing among the three arms of the tripod near the sterically crowded tertiary amine of the ligand, a pivot about a C-N bond results in the inversion of the propeller [83]. Adapted from reference [83].

Systematic exploration by the Canary group of amino acid derivatives [53,82] led to the discovery of a molecule that inverts helicity and ECCD couplet sign upon one-electron redox change [83]. A ligand derived from methionine forms a tetradentate complex with Cu(II) involving three nitrogen atoms and a carboxylate (Figure 18). In this system, the propeller twist of the molecule is dictated by the asymmetric carbon center. Upon reduction to Cu(I), the ligand reorganizes and the sulfide moiety replaces the carboxylate, which requires a pivot about the bond between the tertiary nitrogen atom and the asymmetric carbon atom, inverting the helical orientation of the two quinoline moieties and the exciton chirality spectrum. The switching was reversible with cyclical additions of ascorbate and persulfate. No fatigue was observed after three complete cycles. Crystallographic data supported the structural assignments [52]. Recently, a redox-triggered porphyrin tweezer was reported in an attempt to develop materials with optical properties in the visible region of the electromagnetic spectrum [84]. The Cu(I)/Cu(II) complexes of other tripodal ligands also give inversion of the CD spectrum including derivatives of methioninol and *S*-methylcysteine [85].

Polythiophene oligomers with side chains containing remote chiral centers were prepared by Goto and Yashima *et al*. and studied in solution [86]. The oligomers form chiral aggregates in certain solvents that exhibit induced circular dichroism (ICD). Oxidative doping of the polymer main chain with a Cu(II) salt resulted in the disappearance of CD. Further addition of amines removed the doping, consequently regenerating the ICD signal.

Fukushima and Aida showed an oligomeric o-phenylene that undergoes a redox-responsive dynamic motion [87]. In solution, the helices undergo a rapid inversion, which is dramatically suppressed when it is oxidized by a one-electron oxidant (4-BrC_6_H_4_)_3_N^•+^[SbCl_6_^−^]. X-ray crystallography indicated that a hole, generated upon oxidation, is shared by the repeating *o*-phenylene units, enabling conformational locking of the helix and a long-lasting chiroptical memory.

The activity of a single-site titanium-based lactide polymerization initiator supported by a ferrocenyl-derivatized salen ligand is shown to be modulated by a chemical redox switch; a substantially higher rate of propagation is found for the neutral catalyst compared to its oxidized dicationic ferrocenium counterpart [88].

### 2.5. Electrochemically Triggered Switches

Redox-based chiroptical switches controlled by electrochemical means [50,77] allows for precise tuning and dosing of the required energy input. However, such control is often confined to the interfaces dictated by the electrodes’ surfaces. It also requires the presence of a suitable electrolyte and a polar medium. Cyclic voltammetric measurements of two novel optically active poly(phenylacetylene)s bearing riboflavin (vitamin B2) residues as the pendant through different covalent linkages, revealed that these polymers possess reversible redox-based chiroptical properties originating from the electron transfer process of the riboflavin pendants [89]. The switching of the chiroptical properties in response to the redox stimuli was also investigated using a chemical reductant (Na_2_S_2_O_4_) and oxidant (O_2_).

Although no redox-switchable asymmetric catalysis has been reported, a pioneering demonstration showed that incorporation of a redox-active functionality within a ligand framework potentially allows the reactivity and selectivity to be modulated through either chemical or electrochemical switching of the redox center(s). The hydrogenation of cyclohexene catalyzed by a diphosphino-cobaltocene stabilized rhodium complex (**I**_red_), is much faster than with its dicationic cobaltocenium analogue(**I**ox). The more electron-rich Rh center of **I**_red_ is better able to promote the oxidative addition of H_2_ [90]. There are other similar reports.

## 3. Mechanically Switchable Chiroptical Switches

**Figure 19:**
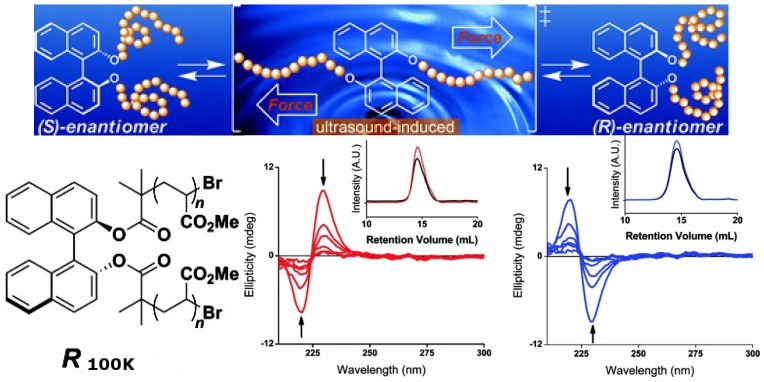
Top: Mechanically facilitated reconfiguration processes of ***R*_100K_** and ***S*_100K_**. Bottom: CD spectra of CH_3_CN solutions of (red) ***S*_100K_** and (blue) ***R*_100K_** (0.1 mg/mL) as functions of time under the sonication. The arrows indicate the direction of the spectra as they changed over time. The insets show gel-permeation chromatograms of ***S*_100K_**and ***R*_100K_**before (red and blue, respectively) and after sonication (black) [91]. Reproduced by permission of the American Chemical Society.

Chiral molecules such as 1,1′-bi-2-naphthol (binol), 2,2′-bis(diphenylphosphino)-1,1′-binaphthyl (binap) and their derivatives, whose asymmetric structures are derived from hindered rotations about sterically congested bonds, have isomerization barriers that typically exceed 30 kcal mol^−1^ and do not readily undergo thermal equilibration. However, as demonstrated recently, thermally restricted isomerization barriers can be surmounted by force [91]. A binol derivative ((*S*)-1,1′-binaphthyl) outfitted with poly(methyl acrylate) (PMA) polymer chains of sufficient molecular weight (***S*_100K_**) was dissolved in CH_3_CN and subjected to sonication. CD analysis of aliquots removed periodically from this solution revealed a smooth decrease in signal intensity over time (Figure 19). After 24 h of sonication, >95% of the material had undergone racemization. Similar results were obtained when the aforementioned experiments were performed with ***R*_100K_**, a PMA polymer embedded with (*R*)-1,1′-binaphthyl. A series of control experiments and extensive characterization showed that such isomerization reactions are consistent with mechanically facilitated processes that proceed with minimal chain scission. Although the result is the racemization of the binol, which cannot be reversed yet, this confirms that planar intermediates are accessible by applying a tensile force to binol derivatives outfitted with polymer chains of sufficient molecular weight, ultimately converting half of the molecules from one enantiomer to the other. One can imagine that the chirality of such molecules can be switched if a unidirectional mechanical force can be applied.

Mechanical forces are unconventional alternative stimuli. Recently, macroscopic mechanical forces have been successfully used to induce chemical transformations. 

## 4. Temperature Triggered Switches

Stimulus encoded in temperature changes can lead to dramatic responses. Subtle conformational changes caused by temperature changes can have dramatic effects on enzyme activity. There are several ways to introduce thermal energy into a system, although a precise control over location and time is hard to achieve.

Sugiyama prepared DNA and RNA based systems that switch between right and left-handed helices in response to temperature stimuli. Although there are distinctive changes in CD, aminopurine (Ap) moieties were incorporated so that the *π*-stack information of the transition from right-handed to left-handed DNA (B-Z transition) and RNA (A-Z transition) can be converted into enhanced fluorescence signals. When the temperature is changed from low to high, the RNA device switches from right-handed to left-handed, turning on fluorescence; however, the DNA device switches from left-handed to right-handed, turning off fluorescence. The responses of these RNA and DNA based devices to temperature stimuli are completely reversible for at least 4 consecutive switching cycles [92].

**Figure 20:**
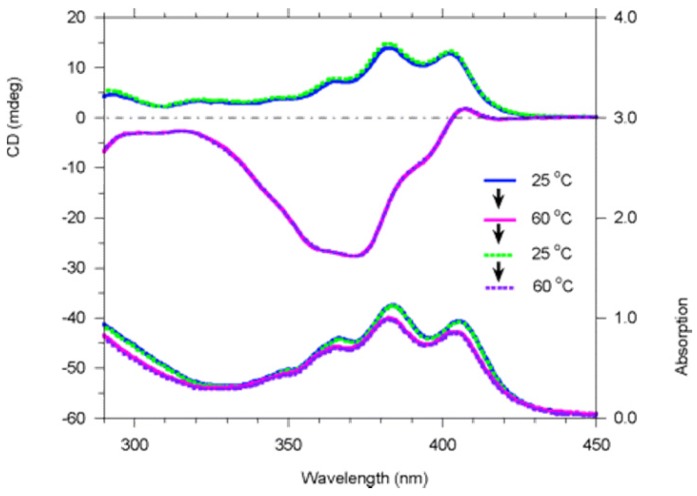
Variable-temperature CD (top) and UV-visible (bottom) spectra of polymer **9** in toluene (*c *= 2.1 × 10^−4^ M, path length = 1 cm) in the heating-cooling-heating thermal cycle [70]. Reproduced by permission of the American Chemical Society.

Full racemization of polymer **9** (structure shown in Figure 14) at +80 °C in toluene requires more than 100 h. Polymer **9** was found to undergo dramatic reversible chiroptical switching that is extremely sensitive and can be driven by heat and solvent polarity. Interestingly, aged (6 months old) polymer **9** was found to undergo fast and completely reversible chiroptical switching at +38.5 °C in toluene in 4 switching cycles that were conducted, the first two of which are shown in Figure 20. Freshly prepared polymer **9** appeared to be not so completely reversible. The helicity of some of poly(propiolic esters) can also be switched thermally [68].

A recently reported *cis*-dichloro sulfur chelated olefin metathesis catalyst was extremely stable at room temperature in solution under ambient conditions and was shown to exhibit a thermo- switchable behavior for ring-closing olefin metathesis of diethyl diallylmalonate, being active at 80 ^○^C and inactive at room temperature [94].

**Scheme 5:**
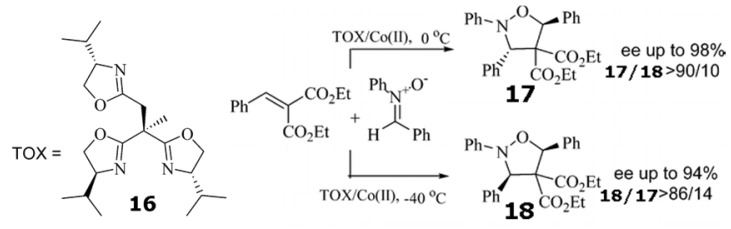
Temperature controls the diastereoselectivity of the 1,3-cycloaddition between nitrones with alkylidene malonates catalyzed by trisoxazoline **16**/Co(ClO_4_)_2_•6H_2_O [93]. Reproduced by permission of the American Chemical Society.

Trisoxazoline **16**/Co(ClO_4_)_2_•6H_2_O catalyzed the 1,3-cycloaddition (Scheme 5) between nitrones with alkylidene malonates at 0 ^○^C to give the isoxazolidines with both high enantioselectivity and high *exo* selectivity. However, when the temperature was lowered from 0 to −40 ^○^C, the same cycloaddition afforded *endo* isomers as the major products with good to high enantioselectivity [93]. On the basis of this experimental evidence, it was concluded that reaction to form *cis*-isoxazolidine is reversible and subject to kinetic control at −40 ^○^C. In the case of the reaction at 0 ^○^C, the cycloaddition is subject to thermodynamic control, favoring the *trans* isomer.

## 6. Photoswitchable Chiroptical Switches

Light arguably represents the most attractive stimulus [3]. A suitable photochrome incorporated in a molecular recognition system enables such a switch to be gated by light of different wavelengths, allowing for the precise control of sensing events and chemical reactivity. Highly precise spatial and temporal control of light irradiation can be easily accomplished, leading to an overall localization and amplification of an optical signal and translation into chemical action.

A planar chiral photoresponsive compound has been employed in commercially available nematic liquid crystals to achieve phototunable reflection colors [95].

**Figure 21:**
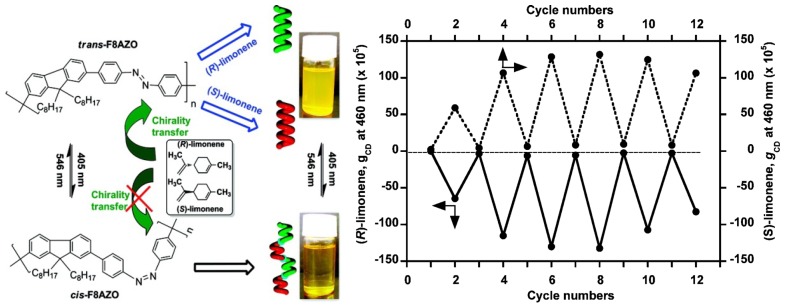
Right: Schematic illustration of unpolarized-light-driven chiroptical switching in CHCl_3_/(*S*- or *R*-limonene)/IPA (0.3/1.5/1.2, v/v/v). Left: Change in the g_CD_ value at 460 nm upon alternating photoirradiation cycles with 405 and 546 nm light [96]. Reproduced by permission of the American Chemical Society.

An achiral polymer, poly[(9,9-di-*n*-octylfluorenyl-2,7-diyl)-alt-4,40-azobenzene] (F8AZO), can undergo reversible *trans*-*cis* photoisomerization upon alternating unpolarized photoirradiation with 405 nm (trans) and 546 nm (*cis*) light (Figure 21). When this polymer is dissolved in enantiomerically pure limonene, the chirality of the solvent allows for the generation of optically active F8AZO aggregates, revealing intense circular dichroism (CD) signals in the visible region. After this solution is irradiated at 405 nm, CD amplitude dropped to almost zero. Upon irradiation at 546 nm, the intense CD signal is restored. The reversible chiroptical response can be repeated for at least 6 switching cycles, although there appeared to be some fatigue in cycles 5 and 6 [96].

Photocontrol of a piperidine’s Brønsted basicity was achieved by incorporation of a bulky azobenzene group and could be translated into pronounced reactivity differences between ON and OFF-states in general base catalysis. This enabled successful photomodulation of the catalyst’s activity in the nitroaldol reaction (Henry reaction) [97]. Although the catalyst involved is chiral, there is no switch in chirality.

**Figure 22:**
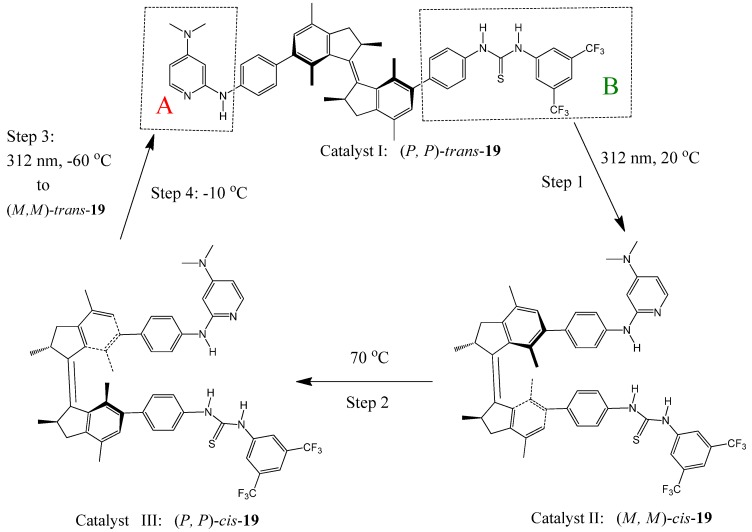
An integrated unidirectional light-driven molecular motor and bifunctional organocatalyst **19** (bottom) [98]. Adapted from reference [98].

Feringa *et al.* recently reported a light-driven molecular motor **19** with integrated catalytic functions [98]. The motor is composed of a rotor A and stator B connected by an alkene axle (Figure 22). Rotor A (DMAP) and Stator B (thiourea) can function as Brønsted base and hydrogen bond donor in an organocatalytic conjugate addition, respectively. Clockwise rotation (seen from the stator side starting from *trans*-**19**) of the rotor around the axle, by photochemically (step 1: 312 nm, 20 ^○^C) and thermally (step 2: 70 ^○^C) induced steps, controls the position and helical orientation of the catalytic groups A and B, providing sequentially catalysts I [(*P,P*)-*trans*-**19**], II [(*M,M*)-*cis*-**19**], and III [(*P,P*)-*cis*-**19**] with different activities and stereoselectivities. Step 3 (312 nm, −60 ^○^C) and step 4 (−10 ^○^C) of the 360 °C rotary cycle reset the catalyst to its initial stage I. They were used to catalyze the Michael addition of 2-methoxy thiophenol to cyclohexenone. When the (*P,P*)-*trans*-**19** (catalyst I) was used, racemic (*R,S*)-thiol adduct was obtained in a slow reaction (7% yield after 15 h). The same reaction proceeds significantly faster (in 50% yield) and enantioselectively [affording a (*S*)-thiol adduct with 50% *ee*] when the (*M,M*)-*cis*-**19**(catalyst II) was used under the same conditions. Catalyst III [(*P,P*)-*cis*-**19**] accelerates the addition reaction even more (83% yield in 15 hours) and exhibits an inversion in enantioselectivity, providing the (*R*)-thiol adduct with 54% *ee*.

## 7. Conclusions

With such a wide scope of systems designed and discovered, chiroptical switches are poised to play expanded roles in many applications including chiral analysis, absolute configurational assignment, sensors, catalysts, and electro-optical displays, modulators, and data storage. Among these applications, their versatile capabilities in the detection of several types of analytes and in the asymmetric catalysis of many types of reactions stand out. The ultimate potential of chiroptical switches in these two fields is extreme. Sensing of biologically relevant species could be incorporated into medical technology aiding disease prevention and treatment. Sensors capable of detecting chiral phenomena on or in extraterrestrial terrain have attracted to search for nonracemic organics outside of the earth’s biosphere, which would offer significant implications regarding the origins of chirality in life and on earth. As we have discussed, not only chiral analytes are of interest but chiroptical spectroscopy and rationale offer unique strategies for detection of a variety of achiral analytes. By virtue of its capabilities to switchably recognize and respond to a host of stimuli, a single chiroptical switch (in most cases an achiral molecule) can be used in controlled catalytic production of either enantiomer or diastereomer at will through selective use of a low-cost inducer: Co-catalysts (guests), metal ions, counter ions or anions, redox agents or electrochemical potential, solvents, mechanical forces, temperature or electromagnetic radiation. Although the maximum number of reversible cycles that these chiroptical switches can be operated are not known, some of them can be reversibly switched multiple times without showing significant fatigue, offering the possibly of easy re-use as sensors or catalysts.
